# Endovascular Graft Suprarenal Bare Metal Stent Separation After Endovascular Aneurysm Repair: Case Reports and Literature Review

**DOI:** 10.1016/j.ejvsvf.2023.06.001

**Published:** 2023-07-19

**Authors:** Jeffrey M.A. van der Krogt, Anne C. van Erp, Stefan P.M. Smorenburg, Martine C.M. Willems, Johanna H. Nederhoed

**Affiliations:** a Department of Surgery, Amsterdam UMC, Location AMC, Amsterdam, the Netherlands; b Amsterdam Cardiovascular Sciences, Amsterdam, the Netherlands

**Keywords:** Aortic aneurysm, Device failure, Endovascular aneurysm repair, Stent graft, Suprarenal fixation

## Abstract

**Objective:**

Suprarenal bare metal stent separation is a rare complication after endovascular aneurysm repair. In this report, two new cases of this type of device failure are presented and the literature is reviewed to identify similar cases and evaluate associated clinical characteristics.

**Methods:**

A literature search was conducted in March 2022 using PubMed, Embase, and The Cochrane Library, with MeSH terms including aortic aneurysm, stents, and device failure. Two authors independently selected studies eligible for inclusion.

**Results:**

Twelve patients with endovascular graft suprarenal bare metal stent separation were identified. Endovascular aneurysm repair (EVAR) devices were implanted between May 1996 and November 2017. Suprarenal bare metal stent separation was detected after a median duration of five years post-operatively.

**Conclusion:**

Endovascular graft suprarenal bare metal stent separation demands a high level of awareness. A better understanding of the involved failure mechanisms and associated risk factors is required to further optimise EVAR follow up protocols.

## Highlights

• Twelve cases of endovascular graft suprarenal stent separation were identified.
• Identified cases concern EVAR devices implanted between 1996 and 2017.
• Suprarenal stent separation was detected after a median duration of five years.
• Early detection of this defect requires improved knowledge on the failure mechanism.

## Introduction

Endovascular aneurysm repair (EVAR) offers a minimally invasive alternative to open repair of abdominal aortic aneurysms (AAAs). Despite a lower overall peri-operative mortality for EVAR compared with open repair, recent studies have demonstrated higher mortality rates for EVAR in the midterm ranging from two to six years after repair.^1^ This phenomenon is partially due to the higher rate of EVAR device failure during this period. EVAR device failure is often multifactorial, including a combination of hostile necks (characterised by a length shorter than 15 mm, large diameter, tapered or reverse tapered anatomy, mural thrombus, moderate or severe circumferential calcification, or angulation), suboptimal pre-operative planning, inaccurate stent graft implantation (e.g., proximity to the lowest renal artery), poor case selection (not respecting the instructions for use [IFU]^2^), stent graft infection, and in rare cases mechanical issues with the stent graft. When left untreated, EVAR device failure may result in endoleaks, an increase in aneurysm sac size and eventually aneurysm rupture.

One type of EVAR device failure particularly seen in the hostile neck with a short seal zone is distal migration of the stent graft. To prevent this issue, various stent grafts have been modified with suprarenal bare metal stent fixation.^3^ Although this modification has led to significantly lower migration rates,^4^^,^^5^ suprarenal fixation increases the risk of endovascular stent graft separation, a situation that requires prompt re-intervention to prevent endoleaks and aneurysm rupture. Here, two new cases of endovascular graft suprarenal bare metal stent separation are reported. Additionally, a literature review is performed on second and third generation stent graft devices with suprarenal fixation to identify similar cases and evaluate associated clinical characteristics.

## Materials and methods

With the aim of identifying cases of suprarenal stent separation, a literature search was conducted in PubMed, Embase, and the Cochrane Library on 22 March 2022. MeSH terms included aortic aneurysm, stents, and device failure. Apart from limiting the search to English literature, no restrictions were placed on article type, year of publishing, or type of aortic stent graft.

During the initial study selection, two authors (J.v.d.K. and A.v.E.) independently screened article title, keywords, and abstract using Rayyan QCRI (Qatar Computing Research Institute^6^). Unmatched decisions were resolved through consensus. Selected studies were subsequently assessed for inclusion based on full text analysis. Primary outcome measures included patient characteristics (sex, age, past medical history), AAA dimensions (AAA diameter, aortic neck length and diameter, aortic angulation, adherence to IFU), EVAR specifications (year of EVAR, originally implanted device, graft dimensions, oversizing), and details of the device failure and re-intervention strategy (time to defect, graft migration, AAA rupture, re-intervention approach, re-intervention device). Extracted data were analysed using Microsoft Excel. Supplementary Appendix A provides an overview of the search methods, search queries, and study selection process.

## Results

### Case reports

The first reported case involved a 78 year old man who presented with heavy pain in his lower abdomen. His medical history included an anteroseptal myocardial infarction, coronary artery bypass grafting, an ischaemic cerebrovascular accident, and a ruptured left common iliac artery with a concomitant 64 mm infrarenal AAA (aortic neck length of 42 mm and neck diameter of 23 mm) for which an emergency EVAR had been performed four years and three months before (Zenith Alpha stent graft, Cook Medical, Bloomington, IN, USA; 28 × 84 mm, left leg 13 × 93 mm extended with a 13 × 59 mm leg, right leg 16 × 77 mm). Due to a 63° aortic neck angulation relative to the infrarenal aneurysm, this procedure was performed just outside the IFU. After EVAR, the patient withdrew from outpatient follow up.

On arrival at hospital, computed tomography angiography (CTA) of the abdomen revealed AAA rupture, accompanied by endovascular graft suprarenal bare metal stent separation with distal graft migration and a type Ia endoleak (Fig. 1). A static 3D reconstruction of this mechanical failure is provided in Supplementary Figure S1. Emergency endovascular relining of the stent graft was carried out with a Gore Excluder AAA endoprosthesis (W.L. Gore & Associates, Inc, Flagstaff, AZ, USA) (28.5 × 14.5 × 12, left leg 14.5 × 12, right leg 14.5 × 10). Completion angiography showed patent renal arteries and iliac limbs (Fig. 2). The patient was discharged six days post-operatively in good clinical condition.

**Figure 1:**
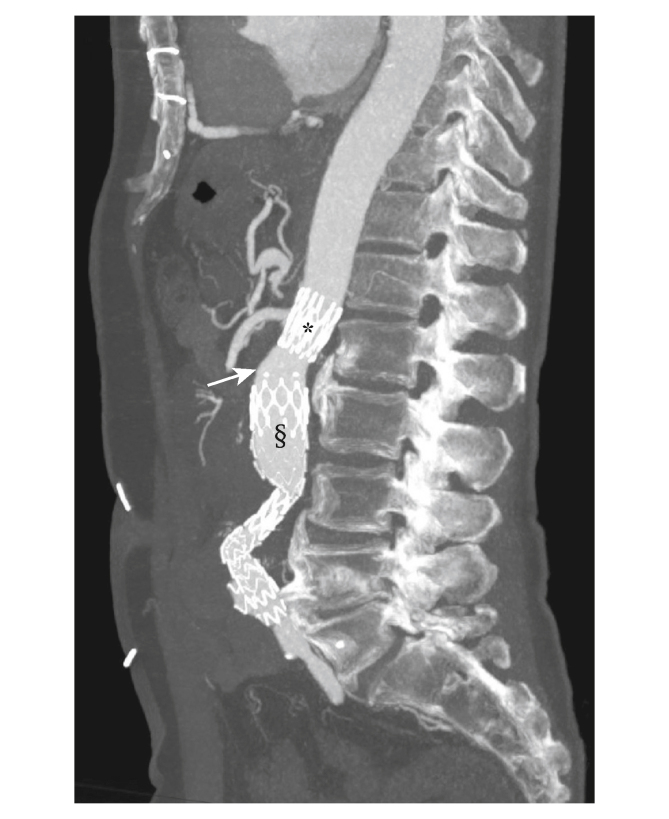
Computed tomography angiogram (sagittal plane) of a 78 year old male patient with abdominal aortic aneurysm showing separation (white arrow) of the suprarenal bare metal stent (∗) from the graft main body (§) after endovascular aneurysm repair.

**Figure 2:**
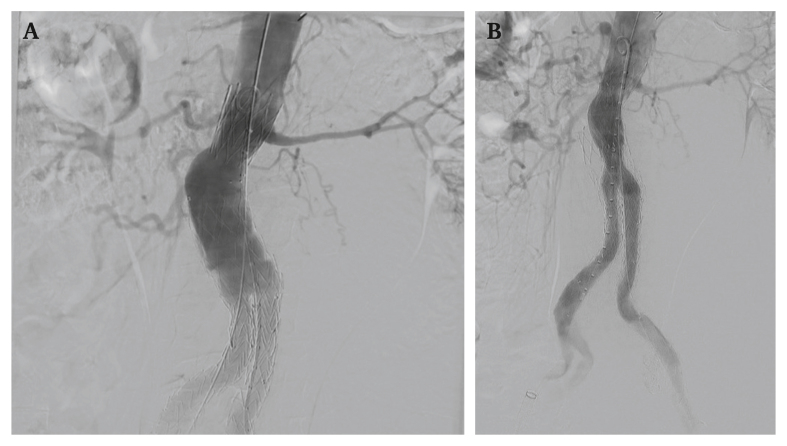
Angiography (coronal plane) of a 78 year old male patient with abdominal aortic aneurysm. (A) The failed endovascular stent graft before relining. (B) The failed endovascular stent graft after relining showing patent renal arteries and iliac limbs.

The second case involved a 74 year old man with a history of arterial calcifications and minimally invasive aortic valve replacement. A 62 mm infrarenal AAA (aortic neck length of 15 mm and neck diameter of 31 mm) was discovered incidentally on follow up CTA of the heart, for which EVAR was performed in January 2016 (Zenith Flex AAA endovascular graft, Cook Medical, Bloomington, IN, USA; 22Fr × 36 × 113 mm, left leg 16 ×74 mm elongated with a 20 × 90 mm leg, combined with a Zenith right sided iliac branched device, Cook Medical, Bloomington, IN, USA; 45 × 41 mm). This procedure was performed outside the IFU as the aortic neck angulation relative to the axis of the suprarenal aorta was measured at 61° whereas an angle smaller than 45° is recommended.

**Figure 3:**
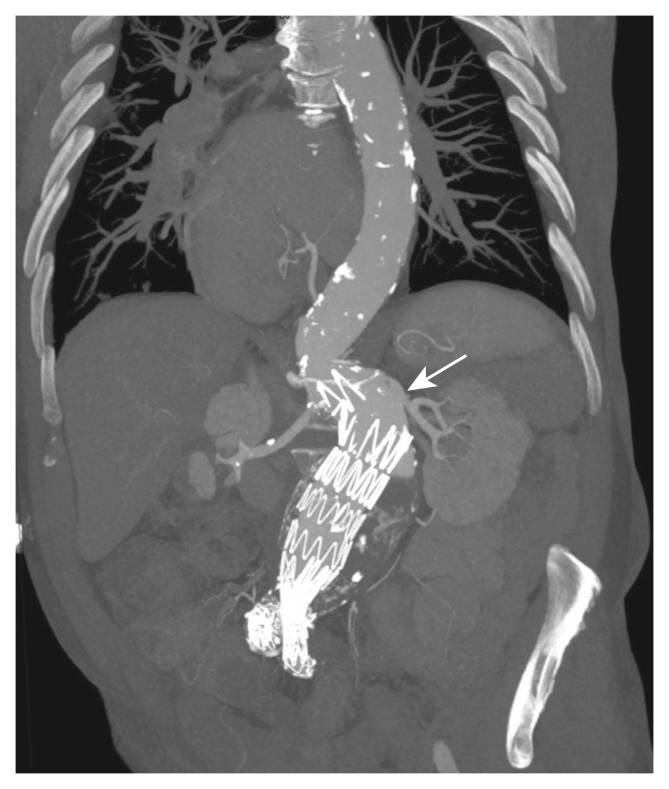
Computed tomography angiogram (coronal plane) of a 74 year old male patient with abdominal aortic aneurysm showing endovascular graft suprarenal bare metal stent separation (white arrow).

Follow up CTA and duplex ultrasound imaging showed a gradual shrinkage of the AAA diameter down to a minimum of 54 mm and no signs of an endoleak during the first four years of follow up. Five years and eight months post-implantation, the patient developed progressive dyspnoea on exertion. During cardiac examination, an enlargement of the AAA sac to 65 mm in diameter was found, accompanied by suprarenal bare metal stent separation and distal graft migration of the endovascular graft leading to a type Ia endoleak (Fig. 3). A 360° angle 3D reconstruction of this mechanical failure is provided in Supplementary Video S1. Because of aortic angulation and technical difficulties raised by the stent graft defect in combination with a fast growing aneurysm, the patient was scheduled for open repair. Unfortunately, surgery was postponed due to the patient's comorbidities, including possible non-small cell lung cancer (cT1aN0M0) and an intrathoracic stomach. The patient died as a result of multiorgan failure induced by abdominal sepsis resulting from intestinal ischaemia before surgery of his aneurysm could take place.

Supplementary data related to this article can be found at https://doi.org/10.1016/j.ejvsvf.2023.06.001.

### Literature search and systematic review

The literature search yielded 529 studies from which nine case reports were included for data analysis.^7^, ^8^, ^9^, ^10^, ^11^, ^12^, ^13^, ^14^, ^15^ In total, 12 cases of endovascular graft suprarenal bare metal stent separation have been described in detail in the literature. All cases involved male patients aged between 67 and 85 years with AAA diameters ranging from 50 to 95 mm. A medical history apart from AAA was reported for seven patients, most commonly involving ischaemic coronary disease (*n* = 4), hypertension (*n* = 4), and chronic obstructive pulmonary disease (*n* = 3).

EVAR procedures were performed between May 1996 and November 2017, with 10 patients receiving a Zenith device (Zenith Alpha^7^^,^^8^ *n* = 3; Zenith Flex^12^ *n* = 2; Zenith Low-Profile^14^ *n* = 2; unspecified^10^^,^^11^^,^^15^ *n* = 3; Cook Medical, Bloomington, IN, USA/William A. Cook, Brisbane, Australia) and two patients receiving a Medtronic device (Talent^13^ *n* = 1; Endurant^9^ *n* = 1; Medtronic, Inc., Minneapolis, MN). Four of seven cases for which complete data on aortic dimensions was provided were performed outside the IFU.

Suprarenal stent separation was detected after a median duration of five years post-operatively. CTA imaging served as the primary diagnostic modality in six cases, whereas in the remaining six cases CTA imaging was used to confirm the detection of suprarenal stent separation by ultrasound duplex or plain radiography examinations. While two patients were completely asymptomatic at detection of suprarenal bare metal stent separation, clinical manifestations were reported for six of 12 patients with abdominal pain as the most prevalent symptom (*n* = 3).

Endovascular graft bare metal stent separation was accompanied by distal migration of the graft main body in all twelve cases. In four of the twelve cases, the pre-existing AAA had ruptured. Re-intervention consisted of endovascular relining in nine patients, while three patients underwent open conversion due to anatomical or mechanical difficulties. Further details on patient characteristics, AAA dimensions, adherence to IFU, and specifications of the original EVAR and re-intervention procedures are provided in Supplementary Tables S1 and S2.

## Discussion

In this study, two new cases of suprarenal bare metal stent separation after EVAR have been reported. Additionally, 10 similar cases were identified in the literature, involving male patients aged between 67 and 85 years with AAA diameters ranging from 50 to 95 mm. Among the 12 cases in total, suprarenal stent separation was detected after a median duration of five years post-operatively. Four of seven EVAR procedures for which complete data on aortic dimensions were provided were performed outside the IFU.

The durability of an EVAR device generally depends on the interplay between the device, the anatomical configuration, and the haemodynamics of the aneurysm. Directly after deployment, the aortic stent graft is subjected to forces in multiple directions, generated by retrograde and antegrade flow and systolic and diastolic blood circulation. In response to several reports of endovascular graft stent separation among first generation EVAR devices,^4^^,^^10^ Zenith stent grafts were modified with double suture reinforcement in 2002 to secure the graft main body to the proximal bare stent. Despite this modification, seven of 12 cases included in the current study involved Zenith EVAR devices implanted after 2002, suggesting that the defect was not fully eliminated by double suture reinforcement. Indeed, in one case report, the explantation of an EVAR device with reinforced sutures directly after suprarenal stent separation revealed intact sutures that had eroded through the graft material.^14^ This finding underscores that, although suprarenal bare metal stent separation occurs sporadically (relative to the annual number of EVAR implantations performed worldwide), further elucidation of the corresponding mechanism of failure is needed to secure EVAR safety.

In addition to the 12 cases of endovascular graft suprarenal bare metal stent separation evaluated in this study, similar cases have been reported in the literature but were not included due to a lack of information on patient and EVAR characteristics.^4^^,^^16^, ^17^, ^18^ Nonetheless, the recurrent mentioning of this issue underscores the need for a follow up strategy that adequately recognises suprarenal stent separation defect at an early stage. Currently, the follow up protocol after EVAR consists of CTA one month after the procedure. In the absence of complications, the patient is followed with ultrasound duplex examination annually thereafter. If there is a complication, CTA will be repeated after six and 12 months or earlier depending on the type of complication. Where this follow up strategy is mostly based on aneurysm characteristics and stent graft related complications, patient specific aortic dynamics (e.g., wall shear stress, blood flow patterns, and force emissions on the stent graft) are not routinely measured. Considering the impact of aneurysm haemodynamics on EVAR device durability, the assessment of aortic dynamics by novel imaging techniques such as 4D flow magnetic resonance imaging, dynamic CTA, and high frame rate contrast enhanced ultrasound may facilitate timely detection of imminent suprarenal stent separation in the future. Moreover, since the European Society for Vascular Surgery guidelines for the management of AAA has advised a stricter surveillance programme after EVAR in patients with a high risk of complications (e.g., EVAR performed outside the IFU and the absence of long term data on durability),^19^ it is of specific interest to further identify risk factors associated with suprarenal stent separation.

### Conclusion

Endovascular graft suprarenal bare metal stent separation is a serious form of device failure that demands a high level of awareness. Improved knowledge on the failure mechanisms and associated risk factors is required for optimisation of stent graft safety after EVAR. In the future, the assessment of patient specific aortic dynamics by novel imaging techniques might facilitate early detection of imminent suprarenal stent separation.

## Conflict of interest

None.

## funding

None.

## Figures and Tables

**figs1:**
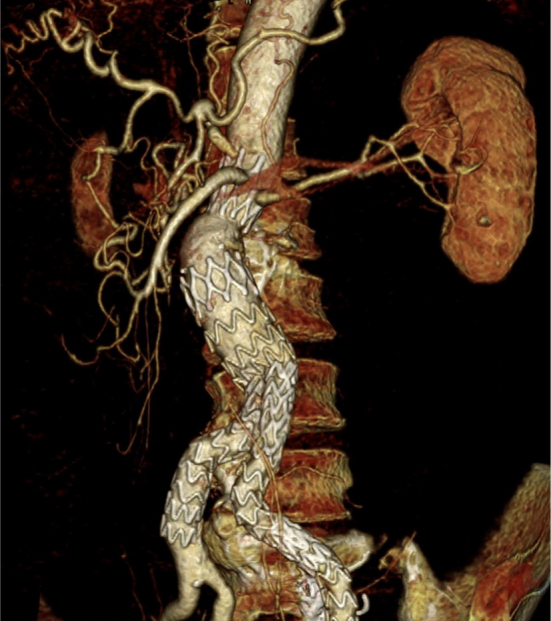


## References

[1] Yokoyama Y., Kuno T., Takagi H. (2020). Meta-analysis of phase-specific survival after elective endovascular versus surgical repair of abdominal aortic aneurysm from randomized controlled trials and propensity score-matched studies. J Vasc Surg.

[2] Hahl T., Protto S., Järvenpää V., Uurto I., Väärämäki S., Suominen V. (2022). Long-term outcomes of endovascular aneurysm repair according to instructions for use adherence status. J Vasc Surg.

[3] Zayed H.A., Bell R.E., Clough R.E., Thomas S., Sabharwal T., Reidy J.F. (2009). Results of endovascular repair of abdominal aortic aneurysms with an unfavorable proximal neck using large stent-grafts. Cardiovasc Intervent Radiol.

[4] Greenberg R.K., Chuter T.A.M., Cambria R.P., Sternbergh W.C., Fearnot N.E. (2008). Zenith abdominal aortic aneurysm endovascular graft. J Vasc Surg.

[5] Abbruzzese T.A., Kwolek C.J., Brewster D.C., Chung T.K., Kang J., Conrad M.F. (2008). Outcomes following endovascular abdominal aortic aneurysm repair (EVAR): an anatomic and device-specific analysis. J Vasc Surg.

[6] Ouzzani M., Hammady H., Fedorowicz Z., Elmagarmid A. (2016). Rayyan—a web and mobile app for systematic reviews. Syst Rev.

[7] Berchiolli R.N., Marconi M., Bargellini I., Bertagna G., Adami D., Mocellin D.M. (2022). An unusual cause of failure in Zenith Alpha abdominal endograft. Eur J Med Res.

[8] Ghaly P., Iliopoulos J., Schlaphoff G., Ahmad M. (2021). Repair of a late endoleak following complete proximal endograft fixation strut separation. J Vasc Surg Cases Innov Tech.

[9] Massara M., Barillà D., Franco G., Volpe A., Serra R., De Caridi G. (2016). An uncommon case of type iii endoleak treated with a custom-made thoracic stent graft. Ann Vasc Surg.

[10] Ghanim K., Mwipatayi B.P., Abbas M., Sieunarine K. (2006). Late stent-graft migration secondary to separation of the uncovered segment from the main body of a Zenith endoluminal graft. J Endovasc Ther.

[11] Torres-Blanco Á, Molina-Nácher V., Sala-Almonacil V., Ortiz-Monzón E. (2016). A rare complication after endovascular aneurysm repair. J Endovasc Ther.

[12] Ueda T., Tajima H., Murata S., Iwata K., Saitou H., Miki I. (2019). An extremely rare complication: abdominal aortic aneurysm rupture caused by migration of a Zenith main body years after repair of the suprarenal stent separation. J Endovasc Ther.

[13] Pitoulias G.A., Mavros D.M., Pappas E.A., Atmatzidis S.K., Papadimitriou D.K. (2012). Chronic contained abdominal aortic aneurysm rupture after suprarenal fixation fatigue fracture. Ann Vasc Surg.

[14] Lindström D., Wahlgren C.M., Sonesson B., Resch T. (2016). Disintegration of the top stent on zenith abdominal aortic stent-grafts. J Endovasc Ther.

[15] Smith J., Joseph S., Thoo C. (2023). Zenith AAA endovascular graft suprarenal bare metal stent separation with graft migration and type IA endoleak. Vascular.

[16] Mertens J., Houthoofd S., Daenens K., Fourneau I., Maleux G., Lerut P. (2011). Long-term results after endovascular abdominal aortic aneurysm repair using the Cook Zenith endograft. J Vasc Surg.

[17] Goodman M., Lawrence-Brown M.M.D., Hartley D., Allen Y.B., Semmens J.B. (2007). Treatment of infrarenal abdominal aortic aneurysms with oversized (36-mm) Zenith endografts. J Endovasc Ther.

[18] Dias N.V., Riva L., Ivancev K., Resch T., Sonesson B., Malina M. (2009). Is there a benefit of frequent CT follow-up after EVAR?. Eur J Vasc Endovasc Surg.

[19] Wanhainen A., Verzini F., Van Herzeele I., Allaire E., Bown M., Cohnert T. (2019). Editor's Choice – European Society for Vascular Surgery (ESVS) 2019 clinical practice guidelines on the management of abdominal aorto-iliac artery aneurysms. Eur J Vasc Endovasc Surg.

