# Supplementary material for: Endovascular Graft Suprarenal Bare Metal Stent Separation After Endovascular Aneurysm Repair: Case Reports and Literature Review
Source: EJVES Vasc Forum. 2023 Jul 19;60:28–32. doi: 10.1016/j.ejvsvf.2023.06.001 (PMC10415614; doi:10.1016/j.ejvsvf.2023.06.001)

Appendix A: specification of the literature search
Literature search performed by J.M.A. van der Krogt and A.C. van Erp

Results March 22, 2022

Database	Results	Full text articles
PubMed	362	347
Embase	626	369
The Cochrane Library	21	17
Total	1,009	733
After removing duplicates	-	529

Search Strategy

A comprehensive search was performed in the bibliographic databases PubMed, Embase.com and the Cochrane Library from inception to March 22, 2022. Search terms included controlled terms (MesH in PubMed and Emtree in Embase) as well as free text terms. The following terms were used (including synonyms and closely related words) as index terms or free-text words: ‘aortic aneurysm’ and ‘equipment failure’ and ‘stent’. The search was performed without date restrictions. Duplicate articles were excluded.

Search Queries

PubMed History March 22, 2022

Search	PubMed Query – March 22, 2022	Items found
#5	#1 AND #2 AND #3 AND #4	362
#4	“bare”[tiab] OR “zenith”[All Fields] OR “endurant”[All Fields] OR “suprarenal”[tiab]	29,891
#3	“stents”[MeSH Terms] OR “stent”[tiab] OR “graft”[tiab]	342,679
#2	“equipment failure”[MeSH Terms] OR “separation”[tiab] OR “failure”[tiab] OR “break”[tiab] OR “detachment”[tiab] OR “disconnection”[tiab]	1,183,440
#1	“aortic aneurysm”[MeSH Terms] OR “aort*”[tiab]	320,154

Embase.com History March 22, 2022

Search	Embase.com Query– March 22, 2022	Items found
#5	#1 AND #2 AND #3 AND #4	626
#4	‘bare’:ti,ab,kw OR ‘zenith’ OR ‘endurant’ OR ‘suprarenal’:ti,ab,kw	37,722
#3	‘stent’/exp OR ‘stent’:ti,ab,kw OR ‘graft’:ti,ab,kw	549,415

Search	Embase.com Query– March 22, 2022	Items found
#2	‘equipment failure’/exp OR ‘separation’:ti,ab,kw OR ‘failure’:ti,ab,kw OR ‘break’:ti,ab,kw OR ‘detachment’:ti,ab,kw OR ‘disconnection’:ti,ab,kw	1,577,054
#1	'aortic aneurysm'/exp OR 'aort*':ti,ab,kw	438,352

The Cochrane Library History March 21, 2022

Search	The Cochrane Library Query– March 22, 2022	Items found
#5	#1 AND #2 AND #3 AND #4	21
#4	“bare”:ti,ab,kw OR “zenith” OR “endurant” OR “suprarenal”:ti,ab,kw	2,288
#3	“stents”[MeSH Terms] OR “stent”:ti,ab,kw OR “graft”:ti,ab,kw	40,949
#2	“equipment failure”[MeSH Terms] OR “separation”:ti,ab,kw OR “failure”:ti,ab,kw OR “break”:ti,ab,kw OR “detachment”:ti,ab,kw OR “disconnection”:ti,ab,kw	117,193
#1	“aortic aneurysm”[MeSH Terms] OR “aort*”:ti,ab,kw	12,757

PRISMA search flow diagram

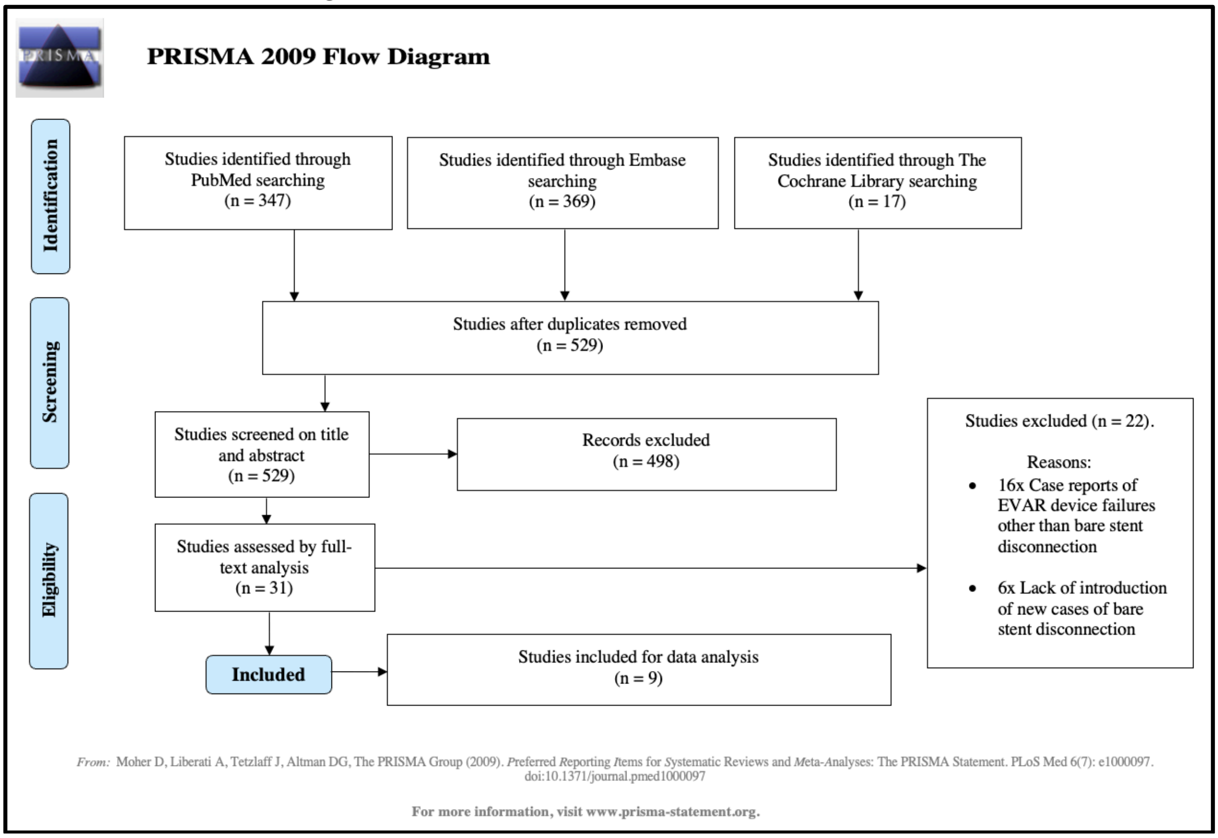

Supplement: Multimedia component 1 [file mmc1.pdf]
