# Supplementary material for: Endovascular Graft Suprarenal Bare Metal Stent Separation After Endovascular Aneurysm Repair: Case Reports and Literature Review
Source: EJVES Vasc Forum. 2023 Jul 19;60:28–32. doi: 10.1016/j.ejvsvf.2023.06.001 (PMC10415614; doi:10.1016/j.ejvsvf.2023.06.001)
Supplement: Multimedia component 2 [file mmc2.pdf]

Appendix B: Data extracted from case reports

Table 1. Patient characteristics and AAA dimensions.

Article	Sex	Age [years]	Past medical history	AAA diameter [mm]	Prox. aortic neck length [mm]	Prox. aortic neck diameter [mm]	Aortic angulation [degrees]	Within IFU? [Yes/No]
Current article; case nr. 1	M	78	Myocardial infarction, ischemic cerebrovascular accident	64	42	23	/IR aneurysm: 63# /SR aorta: 15	No
Current article; case nr. 2	M	74	Arterial calcifications, minimal invasive aortic valve replacement	62	15	31	/IR aneurysm: 55 /SR aorta: 61#	No
Berchiolli, R.N. et al.[7]	M	71	NR	63	16	23	10	Yes
Ghaly, P. et al.[8]	M	78	Atrial fibrillation, hypertension, asthma	95	12#	28	15	No
Massara, M. et al.[9]	M	74	Hypertension, diabetes mellitus, myocardial infarction, COPD, cholecystectomy, mild renal failure	60	NR	28	NR	NR
Ghanim, K. et al.[10]	M	76	Smoking	57	16	20	30	Yes
Torres-Blanco, Á. et al.[11]	M	79	NR	52	24	26	10	Yes
Ueda, T. et al.[12]	M	72	NR	NR	NR	NR	NR	NR
Pitoulias, G.A. et al.[13]	M	85	Sigmoid diverticulitis, arterial hypertension, coronary disease, COPD, diastasis recti	NR	NR	NR	NR	NR
Lindström, D. et al.[14]*; case nr. 1*	M	75	NR	80	10#	NR	45	No
Lindström, D. et al.[14]*; case nr. 2*	M	67	Smoking, myocardial infarction, hypertension, COPD	73	15	NR	22	Yes
Smith, J. et al.[15]	M	77	NR	50	NR	NR	NR	NR

#: outside IFU; /IR aneurysm: relative to infrarenal aneurysm; /SR aorta: relative to suprarenal aorta; AAA: abdominal aortic aneurysm; IFU: instructions for use; NR: not reported.

Table 2. EVAR and reintervention specifications.

**Article; case nr**	**Year of EVAR**	**Originally implanted EVAR device**	**Main body dimensions [mm]**	**Oversizing [%]**	**Time to defect [months]**	**Graft migration? [Yes/No]**	**AAA rupture? [Yes/No]**	**Reintervention approach**	**Applied re-EVAR device**
Current article; case nr. 1	2017	Zenith Alpha†	28 x 84	22	54	Yes	Yes	Endovascular relining	Gore Excluder
Current article; case nr. 2	2016	Zenith Flex†	36 x 113	16	60	Yes	No	Open conversion	NR
Berchiolli, R.N. et al.[7]	NR	Zenith Alpha†	26 x 84	33	24	Yes	No	Endovascular relining	Zenith TX2 thoracic endograft
Ghaly, P. et al.[8]	2015	Zenith Alpha†	36 x 98	29	66	Yes	No	Endovascular relining	Cook custom-made low-profile four-vessel fenestrated device
Massara, M. et al.[9]	2011	Endurant‡	32 x 124	14	48	Yes	No	Endovascular relining	Custom-made thoracic stent graft
Ghanim, K. et al.[10]	1999	Zenith NS*	24 x 70	20	72	Yes	No	Endovascular relining	Zenith stent graft extension
Torres-Blanco, Á. et al.[11]	2006	Zenith NS†	32 x NR	23	96	Yes	Yes	Endovascular relining	Proximal aortic cuff NS
Ueda, T. et al.[12]	2000	Zenith Flex†	NR	NR	156	Yes	Yes	Endovascular relining	Zenith Flex
Pitoulias, G.A. et al.[13]	1996	Talent‡	NR	NR	180	Yes	Yes	Open conversion	Bifurcated Dacron graft
Lindström, D. et al.[14]*; case nr. 1*	2011	Zenith Low-Profile†	NR	NR	24	Yes	No	Endovascular relining	Fenestrated stent graft NS
Lindström, D. et al.[14]*; case nr. 2*	2012	Zenith Low-Profile†	NR	NR	36	Yes	No	Open conversion	Bifurcated Dacron graft
Smith, J. et al.[15]	2012	Zenith NS†	NR	NR	NR	Yes	No	Endovascular relining	Thoracic stent graft NS

†: Cook Medical, Bloomington, IN, USA; ‡: Medtronic, Inc., Minneapolis, MN; *: William A. Cook, Brisbane, Australia; AAA: abdominal aortic aneurysm; NR: not reported; NS: not specified.
